# Molecular properties, including chameleonicity, as essential tools for designing the next generation of oral beyond rule of five drugs

**DOI:** 10.5599/admet.2334

**Published:** 2024-08-27

**Authors:** Diego García Jiménez, Maura Vallaro, Luigi Vitagliano, Lucía López López, Giulia Apprato, Giuseppe Ermondi, Giulia Caron

**Affiliations:** CASSMedChem, Molecular Biotechnology and Health Sciences Dept., University of Torino, Piazza Nizza 44, 10126 Torino, Italy

**Keywords:** bRo5, chameleonicity, ionization, lipophilicity, new chemical modalities, polarity, PROTACs

## Abstract

**Background and purpose:**

The classical drug discovery toolbox continually expands beyond traditional rule of five (Ro5)-compliant small molecules to include new chemical modalities for difficult-to-drug targets. The paper focuses on the molecular properties essential to drive oral bioavailability within the bRo5 framework.

**Experimental approach:**

The first part outlines the concept and methodologies for characterizing bRo5 physicochemical properties, including considerations on chameleonicity; in particular, the paper summarizes the content of the last author’s talk presented during the IAPC-10 Meeting held in Belgrade in September 2023 (https://iapchem.org/index.php/iapc-10-home). The second part of the manuscript presents novel experimental and computational data on three proteolysis targeting chimeras (PROTACs) currently in clinical trials.

**Key results:**

Molecular descriptors of ARV-110, ARV-471, and DT-2216 are reported and the main limitations of the applied experimental approaches are discussed. Moreover, a simple computational method shows how predicting the presence of chameleonic effects.

**Conclusion:**

A full complete physicochemical characterization of three degraders in clinical trials is reported to highlight the differences in physicochemical descriptors between PROTACs dosed orally and intravenously.

## New chemical modalities: beyond traditional rule of five and their peculiar properties

As the new millennium approached, breakthroughs in genomics and human biology unveiled a plethora of potential drug targets, including genes and proteins that were previously unknown or deemed "undruggable" using conventional methods [1]. This offered an opportunity to propel small molecule drug discovery forward, breaking free from traditional targets. Additionally, it fostered the development of novel chemical modalities (NCMs) bridging classical medicinal chemistry and biotechnology [2,3]. NMCs include biologics (*e.g.*, biotech-based products such as RNA-based therapeutics and antibody-drug conjugates) and small molecules that are not Ro5 compliant and encompass both linear and cyclic compounds. Examples of linear derivatives are targeted protein degraders (TPDs), which include proteolysis targeting chimeras (PROTACs) [4], whereas (MCs) and cyclic peptides (CPs) are representative of cyclic derivatives. PROTACs consist of two linked small molecules: one binds to the target protein of interest (POI), and the other binds to an E3 ligase. TPD technology leverages the ubiquitin-proteasome system to degrade undesirable proteins. MCs are generally defined as organic molecules which contain a ring of at least 12 heavy atoms [5]. CPs are small (MW less than 1500 Da) peptides cyclized to improve their pharmacokinetic (PK) profile [6].

**Figure 1.**
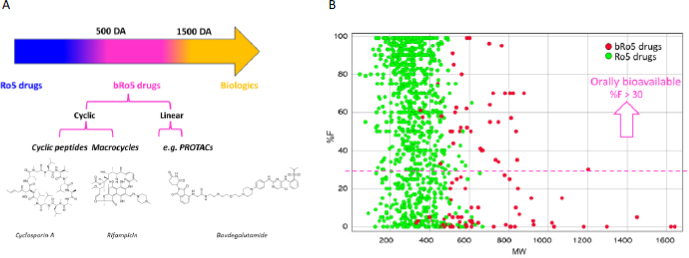
(A) The bRo5 chemical space classification with three representative compounds, the cyclopeptide cyclosporin A, the macrocycle rifampicin and the PROTAC bavdegalutamide (ARV-110). (B) Plot built using data taken from Zhu *et al.* [8] and demonstrating the feasibility of discovering oral bioavailable PROTACs.

PROTACs, MCs, and CPs occupy a chemical space known as the beyond-Rule-of-5 (bRo5) space, characterized by a molecular weight (MW) ranging from approximately 500 to 1500 Da (Figure 1A). This space is situated between Ro5-compliant drugs and biologics. Notably, an increase in MW often correlates with reduced solubility and membrane permeability. This reduction is also linked to the tendency of high-MW molecules to trigger efflux pumps, such as P-glycoprotein (P-gp), which expel drugs from cells [7].

bRo5 molecules can show either high lipophilicity (*e.g*., macrocycles) or high polarity (*e.g.*, cyclopeptides). High polarity, as indicated by metrics like hydrogen bond donors (HBDs), hydrogen bond acceptors (HBAs), and topological polar surface area (TPSA), can enhance solubility but reduce cell permeability. Conversely, high lipophilicity promotes cell permeability but hampers solubility. Additionally, excessively high lipophilicity, often quantified by *c*logP, where *c* indicates any calculation method, can lead to NMCs include retention, compromising oral bioavailability and increasing metabolic clearance and overall toxicity. Achieving an optimal balance between polarity and lipophilicity is therefore challenging for bRo5 derivatives.

bRo5 compounds often show a flexible structure. Notably, high flexibility complemented with structural complexity confers to some bRo5 compounds the ability to adopt different environment-dependent conformations. Molecular flexibility is typically expressed as the number of rotatable bonds (NRot). However, the Kier flexibility descriptor PHI is the only general quantifier that remains valid even when macrocyclic substructures are present [9].

In summary, bRo5 compounds exhibit distinctive molecular characteristics that undermine the efficacy of traditional property-based drug discovery approaches tailored for Ro5 molecules [10]. However, some bRo5 drugs have shown satisfactory oral bioavailability and have been approved for oral formulations (Figure 1B) [11].

## Chameleonicity should be introduced in the pool of bRo5 molecular properties

It has been shown that a few bRo5 compounds have the ability to alter their conformations and molecular characteristics depending on the surrounding environment. This phenomenon, initially observed by Carrupt and colleagues in 1991 [12], was termed "chameleonicity". Formally defined, chameleonicity refers to a compound's capacity to adopt open and polar conformations in aqueous environments while assuming folded and less polar conformations in nonpolar environments such as cellular membranes. In practice, chameleonicity arises from conformational changes that modify the molecular properties of a compound in an environment-dependent manner.

The first and most well-known chameleonicity mechanism is the formation of dynamic intramolecular hydrogen bonds (dIMHBs). In 2011, Alex *et al.* [13] conducted a study analysing the experimental conformations of cyclosporin A (CsA) derived from X-ray crystallography and NMR spectroscopy . In water, CsA forms hydrogen bonds (HBs) with the solvent, adopting an open and more polar conformation. In nonpolar solvents, the HBs are established within the CsA structure, resulting in a closed and less polar conformation. Consequently, the formation of these dIMHBs (dynamic since formed only in the nonpolar environment) resulted in a conformational change from an open conformation in the cytosol to a closed conformation within the membrane interior. It was postulated that this chameleonic behaviour could be the key factor underlying the unexpectedly high permeability of CsA [13]. In practice, CsA is the first well-understood example of a bRo5 drug possessing adequate solubility and cell permeability to transition into an oral medication through molecular chameleonicity.

**Figure 2.**
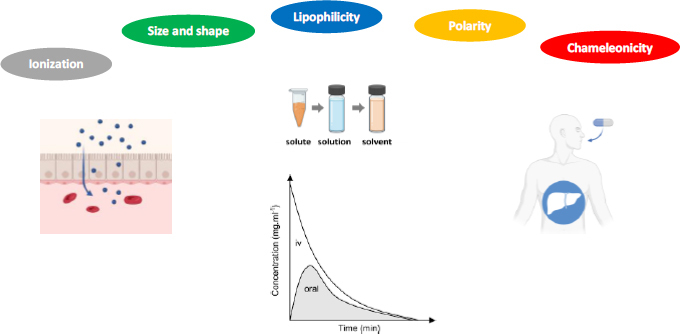
The bRo5 chemical space oral bioavailability determinants: molecular and *in vitro* ADME properties.

Solubility, permeability and metabolic stability are *in vitro* ADME properties that impact oral bioavailability (Figure 2). These latter are in turn modulated by physicochemical properties like ionization, size and shape, lipophilicity and polarity. According to the previous discussion, chameleonicity deserves to be included in the pool of investigated physicochemical properties to predict *in vitro* ADME properties and bioavailability (Figure 2).

## The pool of physicochemical descriptors suitable for the bRo5 space

**Figure 3.**
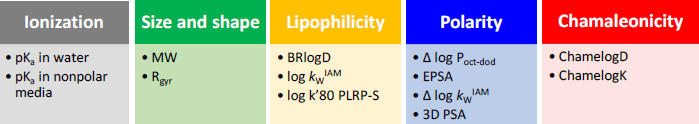
Molecular properties and related descriptors to be applied in the bRo5 space.

Physicochemical descriptors numerically quantify molecular properties. Figure 3 summarizes the physicochemical descriptors, mostly experimental, implemented in our lab to quantify ionization, size and shape, lipophilicity, polarity and chameleonicity.

### Ionization

The ionization constant (usually reported as p*K*_a_) is an important parameter for evaluating ionizable molecules since it may have a decisive impact on the *in vitro* ADME properties. For instance, an ionized species is more water soluble but has a lower cell permeability than the neutral form. p*K*_a_ is often determined in water, but p*K*_a_ measurements in different media can be relevant since p*K*_a_ varies with the media, and thus, a compound may be mostly ionized in water and not ionized in the interior regions of membranes. Recent studies revealed that ionization decreases for both acids and bases in a coherent, significant but not dramatical extent when some amount of an organic solvent (*e.g*. acetonitrile) is present in the environment, but the picture is completely different in pure acetonitrile (MeCN) [14]. Since the experimental determination of p*K*_a_ is not a trivial assay and a certain degree of expertise is required, Caron’s group observed that the ionization behaviour (not the p*K*_a_) of a molecule can be obtained by monitoring the variation of the log of the capacity factor in the PLRP-S system (log *k*'80 PLRP-S) *vs.* the pH [15]. In practice, this variation reveals the acidic, neutral or basic nature of the investigated compound. While measuring p*K*_a_ using standard methods is generally preferable, this estimation can serve as a satisfactory alternative in many instances, especially for compounds like bRo5 molecules that face solubility limitations. Interestingly, and in line with existing literature, the ionization behaviour of bRo5 molecules is rarely experimentally determined. In many cases, ionization is completely overlooked, and in some instances, it is predicted using standard calculators not designed for large, flexible molecules.

### Size and shape

As previously mentioned, bRo5 compounds are known for their large size (MW > 500 Da), which can confer poor solubility and/or permeability across the membrane [16]. Similarly, the efflux pump (such as Pgp), which expels drugs from cells, is activated by molecules with high molecular weight. Additionally, the Stokes-Einstein equation suggests that the shape of the molecule, indicated by the hydrodynamic radius, affects the passive diffusion coefficient [17]. However, the influence of the shape on molecular properties and, thus, on *in vitro* assays is not experimentally clear. There is, therefore, a clear need to computationally quantify these two properties. MW is the default 1D descriptor of molecular size, while the radius of gyration *R*_gyr_ describes the 3D shape of the molecular conformers; the lower the *R*_gyr_, the higher the molecular sphericity. *R*_gyr_ is calculated as the root-mean-square distance (RMSD) of each atom in the molecule from the center of mass using various software programs [18].

### Lipophilicity

Lipophilicity is formally defined by the IUPAC as the affinity of a molecule for a lipophilic environment. Testa and coworkers deconvoluted lipophilicity into a positive contribution of hydrophobicity (water repulsion) and a negative contribution of polarity (separation of electronic charges) [19]. Thus, hydrophobic effects (hydrophobic and steric interactions, hydrophobic collapse, polar group shielding, etc.) increase the lipophilicity of a molecule, whereas the opposite is true for polarity. This implies that lipophilicity and hydrophobicity are not interchangeable terms and that polarity does not stand in opposition to lipophilicity.

The lipophilicity of a compound is expressed as log *P* for neutral derivatives. However, in the case of ionizable centres in the molecule, lipophilicity is expressed as log *D* at a specific pH. Besides the traditional shake-flask method now automated in most Contract Research Organization (CRO) protocols, other methods for measuring lipophilicity, such as potentiometry or high-performance liquid chromatographic-based methods (HPLC), have been reported. The potentiometric method is based on the quantification of the p*K*_a_ change after the addition of octanol to an aqueous solution. HPLC-based methods have gained popularity due to their high degree of automation and their ability to deal with impurities and low concentrations. In this context, the lipophilicity of a certain molecule is measured as the capacity or retention factor (*k*', more often expressed as log *k*') on a certain reverse-phase column (RP-HPLC) at a certain mobile phase composition. Formally, *k*' is defined as the difference between the retention time of the analyte (*t*_R_) and the dead time of the column (*t*_0_), divided by the dead time itself (*t*_0_), equation 1:



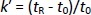

(1)


Under reverse phase (RP)-HPLC conditions, the compounds are retained proportionally to their partition coefficient (generally in the octanol/water system). The use of RP-HPLC for lipophilicity has been exploited in the last two decades by various academic groups and companies, mainly to provide octanol/water surrogates. Valko and coworkers developed the chromatographic hydrophobicity index (CHI) at GlaxoSmithKline (GSK) [20], Pfizer developed its own lipophilicity descriptors (ElogP or ElogD) [21,22] and we developed BRlogD [23]. It should be emphasized that chromatographic methods do not directly measure log *D* and thus they require careful validation in terms of the balance of intermolecular forces governing the retention that should be the same than those governing partitioning in a biphasic octanol/water system. This validation can be obtained by different strategies, *e.g*., the Abraham’s descriptors [24] and/or the Block Relevance (BR) analysis method [25].

log *P*/ log *D*_oct_ and their chromatographic surrogates are popular to model the “average” lipophilicity of compounds in the complex membrane bilayer environment, but log *P*_alk_ (alkane/water) and log *P*_tol_ (toluene/water) better mimic the polarity of the membrane interior (dielectric constant ~2). Also, in this case, chromatography provides surrogates thanks to the wide variety of columns and eluents available. For instance, the PLRP-S (polystyrene/divinylbenzene) is a polymeric column that mimics the alkylic chains of phospholipids. In this context, our group revealed that the capacity factor at 80 % acetonitrile (log *k*' 80 PLRP-S) can be a rough surrogate of the log *P*_tol_ [26].

Furthermore, RP-HPLC methods generate biomimetic environments. For example, immobilized artificial membrane (IAM) chromatography, first introduced by Charles Pidgeon and his colleagues [27], is used to simulate biological cell membranes. IAM columns consist of a monolayer of phosphatidylcholine covalently bound to an inert silica support . The reference descriptor is the logarithm of the capacity factor at 0% organic solvent (extrapolated value), named log *k*_W_^IAM^. In the IAM system, the retention of charged molecules also depends on the electrostatic interactions with the phospholipid heads [28]. Generally speaking, it has been found that IAM columns strongly retain positively charged compounds as the IAM columns are negatively charged on the surface, like cell membranes.

Overall, we recommend measuring lipophilicity in multiple systems to fully assess the lipophilic behaviour of bRo5 compounds, as these molecules are likely to change their conformation and, consequently, their lipophilicity depending on the environment.

### Polarity

While generally less important than hydrophobicity in defining lipophilicity, the significance of polarity varies depending on the system being considered. Experimentally, it can be measured by chromatographic methods. Up to now, two experimental polarity descriptors have been used in the bRo5 space: EPSA and Δ log *k*_w_^IAM^.

The EPSA method has been developed in Pfizer [29,30]. This is a supercritical fluid chromatographic (SFC) method that employs a supercritical fluid as the mobile phase (typically CO_2_) and a normal phase column (Chirex 3014) as the stationary phase. It displays a balance of lipophilic and polar attributes that efficiently separates compounds according to their polarity. In this system, the polarity of the eluent is modified as a gradient by varying the percentage of methanol in an ammonium formate solution. This generates a nonpolar system that favours folded conformations, thereby allowing the determination of a polarity index, designated EPSA, and the monitoring of the presence of IMHBs.

Δ log *k*_w_^IAM^ was developed in 1997 by Barbato and colleagues [28] and is based on the evidence that log *k*_w_^IAM^ (see above) also includes the contribution of the electrostatic interactions between ionized compounds and the phospholipid heads, not detectable by descriptors related to biphasic systems, like octanol/water and toluene/water. Without entering the method details that can be retrieved in the literature [28,31,32], Δ log *k*_w_^IAM^ represents the polarity of the analyte.

**Figure 4.**
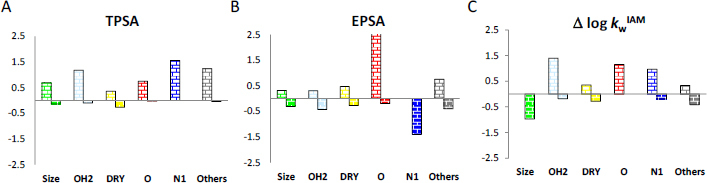
BR analysis (a computational tool that allows the interpretation of the balance of intermolecular interactions governing systems, on the Y axis the value of the block, on the X axis the block) for polarity descriptors. (A) TPSA, (B) EPSA and (C) Δ log *k*_w_^IAM^.

EPSA and Δ log *k*_w_^IAM^ offer distinct pieces of information. This is explained in Figure 4, which compares the Block Relevance (BR) graphical output obtained for the three polarity descriptors: the topological polar surface area (TPSA), EPSA and Δ log *k*_w_^IAM^ [32,33].

In practice, the BR analysis is a computational tool that allows the interpretation of the balance of intermolecular interactions governing systems, including chromatographic ones. Since TPSA is the most obvious polarity descriptor, EPSA and Δ log kwIAM are expected to show BR plots similar to TPSA. However, this is not completely verified for EPSA since HBD and HBA blocks (red and blue, respectively) have opposite signs, while the reverse is true for TPSA and Δ log *k*_w_^IAM^. In practice, the HBD properties of the molecule can be underestimated by EPSA when the structure includes multiple HBA groups.

Polarity is often computationally quantified by the polar surface area (PSA). Several methods have been suggested to calculate the 3D molecular polar surface area (3D PSA), but they rarely reach a consensus. In practice, for the sake of simplicity, we prefer to choose a 3D PSA descriptor comparable to the 2D topological PSA (TPSA). To reach this aim, we adopt the following pair of tools (see Methods): TPSA calculated by AlvaDesc and the 3D PSA calculated with Vega ZZ (probe radius 0 Å).

In a recent paper, Price *et al.* [34] - who also established a new high-throughput method to measure the EPSA named HT-EPSA [35] - introduced ETR, the EPSA-to-topological polar surface area (TPSA) ratio. The researchers suggest that ETR in combination with AB-MPS (a multiparametric scoring function including log *D*, the number of rotatable bonds, and the number of aromatic rings [36]) identifies unique subsets of bRo5 and PROTACs, enabling specialized drug design strategies for improved absorption.

Finally, it should be mentioned that the compound's ability to form IMHBs is strictly related to polarity. To experimentally assess the presence of IMHBs, the difference between the log *P*/*D* in octanol/water and the log *P*/*D* in toluene/water systems can be used and Δ log *P*_oct-tol_ has been validated as an optimal strategy to reveal IMHB formation [37]. However, the poor solubility of bRo5 molecules in toluene hinders its application to the bRo5 space. Moreover, EPSA could also provide information on IMHB formation, given that suitable pairs of compounds are available.

### Chameleonicity

Few tools are available to determine chameleonicity, all with important limitations. The most straightforward method is to compare the X-ray crystallographic conformations of the compound crystallized in both polar and nonpolar solvents [38]. This allows to identify structural indicators of the chameleonic effect by monitoring polarity and shape variations. However, molecular structures are often crystallized in the same solvent due to solubility limitations, which is suboptimal for studying dynamic behaviours. Moreover, X-ray-based chameleonicity analysis is susceptible to the "crystal packing effect," where crystallized conformations may not accurately represent those in solution.

Nuclear magnetic resonance (NMR) serves as a more sophisticated approach, relying on the generation of molecular constraints extracted from the analysis of nuclear overhauser effects (NOE) and proton coupling constants (J coupling). NOE and J coupling data form the basis for the NMR analysis of molecular flexibility in solution (NAMFIS, a widely used NMR data analysis method). Through NAMFIS, the NMR-derived constraints are fitted with a series of computationally generated conformations, allowing the extraction of relative population distributions in solution [39]. This strategy has the advantage of focusing on actual solution conformers, but it is time-consuming, suffers from solubility issues, and, in some cases, overfitting can bias the results and require skilled training, making it inappropriate for early drug discovery.

**Figure 5.**
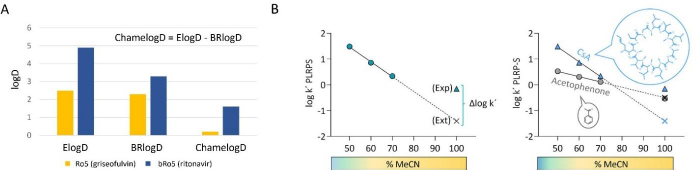
Chameleonicity descriptors (A) ChamelogD for griseofulvin (yellow bars, a nonchameleonic drug) and ritonavir (blue bars, a chameleonic drug) and (B) Chamelogk plots of cyclosporin (CsA, bRo5, blue triangles) and acetophenone (Ro5 compound, gray circles); Exp. log *k*' 100 PLRP-S and Ext. log *k’* 100 PLRP-S values are presented as colored symbols and colored crosses, respectively.

Two HPLC methods have been described up to date to assess chameleonicity (Figure 5): ChamelogD and Chamelogk. ChamelogD (Figure 5A) is the difference between two descriptors ElogD and BRlogD, both surrogates of log *D*_oct_ [23]. Since ElogD and BRlogD are obtained in two different environments, their difference, if any, is related to chameleonicity.

ChamelogD represented the first HPLC-based chameleonicity descriptor suitable for high-throughput (HT) contexts. However, its practical application is complicated by the utilization of two distinct chromatographic systems.

Therefore, we recently implemented a chromatographic descriptor, Chamelogk, that measures chameleonicity in a unique and dynamic system [40]. To obtain Chamelogk (Figure 5B), the first step involves the experimental determination of the capacity factor (log *k*' PLRP-S) at 50, 60, and 70 % of acetonitrile. Then, the linear correlation between the log *k*' PLRP-S and the percentage of MeCN is determined. The equation derived from the linear correlation is then applied to extrapolate the capacity factor at 100 % (called Ext. log *k’* 100 PLRP-S). Then, the experimental value at 100 % MeCN is measured (named Exp. log *k*’ 100 PLRP-S) and the difference with the extrapolated value is calculated. Thus, Chamelogk is defined as the capacity factor difference (Δ log *k*') between the experimental log *k*' measured at 100 % MeCN (Exp. log *k*' 100) and the extrapolated value (Ext. log *k’* 100), as shown by Equation 2 and Figure 5B.



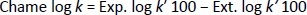

(2)


Experimental determination of the aforementioned physicochemical descriptors is a crucial step in lead prioritization and optimization. However, it is also of great importance to be able to predict the chameleonnicity of a molecule in the early stage of drug discovery, potentially even before synthesis.

**Figure 6.**
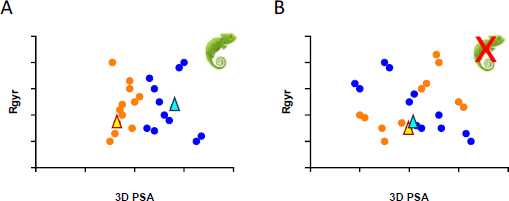
Chameleonicity prediction as obtained from the 3D PSA *vs*. *R*_gyr_ plot: conformers (dots) obtained from CS are coloured according to the environment in which they have been built (blue water, orange nonpolar media) and the minimum energy conformers (triangles) as well (light blue water, yellow nonpolar media). A) a compound with chameleonic properties and B) a non-chameleonic compound.

According to Kihlberg [41] and others [38], a compound to display chameleonic properties (mainly IMHB-driven) should fulfill two requirements: a) to display a broad molecular property space and b) provide privileged polarity regions in an environment-dependent manner (highest polarity in polar environments and minimal in nonpolar ones). Based on these observations, it is clear that chameleonicity cannot be predicted using simple 2D descriptors such as HBD, HBA, TPSA, and PHI. Instead, it requires a) generating an ensemble of conformers and b) characterizing them with appropriate descriptors. In previous papers, we first used conformational sampling (CS) strategies to obtain an ensemble of conformers with their associated energies. Once the conformer populations have been obtained, we monitored conformer properties by calculating polarity (computationally measured by 3D PSA) and spherical shape (measured with a variation of *R*_gyr_). Then, we plot *R*_gyr_ *vs.* 3D PSA, as schematically summarized in Figure 6.

Figure 6A describes the behavior of a compound showing chameleonic properties: the orange dots are the conformers obtained in the nonpolar environments, whereas the blue dots are those extracted from the CS run in water. According to the definition of chameleonicity, the orange dots should show lower polarity than the blue dots. On the other hand, Figure 6B, representing a non-chameleonic behavior, shows that no difference can be monitored by the two pools of conformers. A similar reasoning holds for the minimum energy conformers (MECs) (triangles) (yellow and blue triangles, respectively). However, chameleonicity is a property related to the dynamic behavior of molecules, which is best captured by considering the entire pool of conformers generated in the two environments. For this reason, in some cases, the combined use of CS and molecular dynamics simulation is suggested [42].

## Case studies

**Figure 7.**
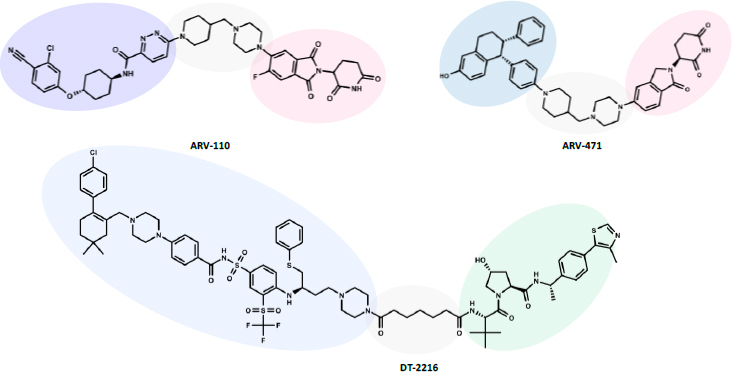
Chemical structures of the investigated PROTACs: the POI ligand is in blue circles, the E3 ligase ligand is in pink (CRBN) and in green (VHL).

Below, we apply all the experimental and computational protocols described above to three PROTACs that have entered clinical trials and have not yet been studied: ARV-110, ARV-471, and DT-2216 (chemical structure in Figure 7).

ARV-110, bavdegalutamide, is the first PROTAC that entered the clinics, and it is currently in phase II clinical trials for the treatment of metastatic castration-resistant prostate cancer (mCRPC, NCT03888612). ARV-110 is an orally bioavailable CRBN degrader targeting AR (androgen receptor) and developed by Arvinas, Inc. Its efficacy was demonstrated both *in vitro* (DC_50_ around 1 nM in all tested prostatic cancer cell lines) and *in vivo*, where it significantly inhibited the growth of enzalutamide-insensitive tumours [43]. In 2019 ARV-110 entered phase I, where its safety, tolerability and pharmacokinetics were evaluated in mCRPC-positive patients not responding to traditional pharmacological treatment. Due to the promising results reported in pretreated mCRPC patients, it entered phase II for further evaluation.

ARV-471, also known as vepdegestrant, is an orally bioavailable CRBN-recruiting ER (estrogen receptor) targeting degrader currently in phase III clinical trials for the treatment of ER-positive/HER2 (human epidermal growth factor 2) negative locally advanced or metastatic breast (VERITAC-2, NCT05654623). Arvinas and Pfizer are collaborating for the co-development and co-commercialization of ARV-471, which showed promising results both *in vitro* (ER degradation up to 97 % in tumor cell lines) and *in vivo* models. ER degrader was well tolerated across all doses orally administered and showed clinical activity in advanced breast cancer patients during phase I and II [44].

DT-2216 is a VHL intravenous PROTAC targeting Bcl-xL (B-cell lymphoma extra-large) for the treatment of solid tumors and hematologic malignancies commercialized by Dialectic. In preclinical studies, DT-2216 selectively induced degradation of Bcl-xL in cancer cells [45]. It is currently in phase I clinical trials to evaluate adverse events, dose-limiting toxicity, and the pharmacokinetic profile (NCT04886622). In contrast with ARV-110 and ARV-471, characterized by a quite high oral bioavailability (*F* > 30 %, preclinical models), DT-2216 is characterized by a very low oral bioavailability (*F* < 0.03 %, mouse). While both ARV-110 and ARV-471 are CRBN-based PROTACs, DT-2216 is a VHL-based degrader (Figure 7).

First we calculated 2D descriptors for the considered PROTACs (Table 1). DT-2216 is characterized by a quite different physicochemical properties profile compared to ARV-110 and ARV-471, and as expected, it can be localized far from the FDA-approved small molecule and CRBN oral PROTACs in the chemical 2D space [46]. DT-2216 is indeed larger, more hydrophobic, more polar, and more flexible than ARV-110 and ARV-471.

**Table 1.** 2D physicochemical descriptors calculated using AlvaDesc and Datawarrior (see Methods). Size descriptors: MW: molecular weight; nC: the number of carbon atoms in the molecule. Flexibility descriptors: PHI: Kier’s flexibility index and nRot: the number of rotatable bonds. Polarity descriptors: HBD: number of hydrogen bond donors, HBA: number of hydrogen bond acceptors and TPSA: topological polar surface area.

Compound	MW	nC	PHI	nRot	HBD	HBA	TPSA
ARV-110	812.38	41	11.96	9	2	16	182.86
ARV-471	723.99	45	9.87	7	2	9	96.43
DT2216	1542.56	77	27.58	29	5	23	321.37

A recent paper by Arvinas researchers [47] reported constraints on PROTAC 2D physicochemical calculated descriptors associated with a higher probability of being orally absorbed. Specifically, a distinct cut-off was observed for HBD, which DT2216 does not meet. Additionally, the same PROTAC also exceeds the HBA threshold of 15.

**Figure 8.**
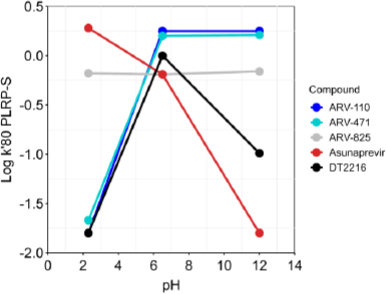
Ionization behavior of the investigated compounds: DT2216, ARV-110 and ARV-471 (ARV-825 and asunaprevir are reported as examples of nonionizable and acidic bRo5 compounds, respectively).

Then, we focused on experimental descriptors. We first explored the ionization behavior of the three PROTACS by monitoring the variation in log *k*'80 PLRP-s at three pHs. Figure 8 shows that ARV-110 (blue) and ARV-471 (light blue) are basic compounds since the logarithm of the capacity factor and, thus, retention increases when passing from acidic to neutral and basic pHs. Moreover, these two PROTACs can be considered predominantly neutral at pH 7. Conversely, DT2216 is an ampholyte since it shows low retention at extreme pHs and the highest retention at pH 7.0. The complex ionization profile of DT2216 prevented the measurements of Chamelogk and log *k*_w_^IAM^ since these methods are not validated for ampholytes.

Experimental lipophilicity (BRlog*D* and log *k*_w_^IAM^) was measured (Table 2). ARV-110 was found to be slightly more polar and less lipophilic than ARV-471. Notably, both PROTACs seem to have a rather similar BRlog*D* and Δlog *k*_w_^IAM^ profile, revealing a similar physicochemical behaviour (despite their different calculated polarity, TPSA). In addition, both compounds behave as molecular chameleons (Chamelog *k* > 0.6), being ARV-471 more chameleonic. On the other hand, DT2216 has a BRlogD over 6, which confirms its extremely high lipophilicity.

**Table 2.** Physicochemical descriptors obtained by chromatographic data. A/B: acid or base profile. ND: not determined.

Compound	Absorption	A/B	BR log D	log *k*_w_^IAM^	Δ log *k*_w_^IAM^	Chamelogk
ARV-110	Oral	Base	4.25	3.20	0.33	0.99
ARV-471	Oral	Base	4.73	3.39	0.07	1.35
DT2216	Intravenous	Ampholite	> 6	> 4	ND	ND

**Figure 9.**
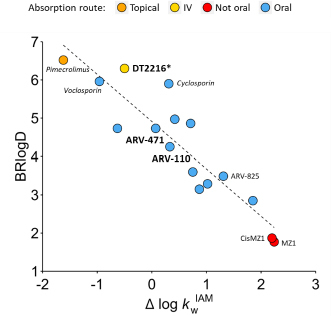
Polarity and lipophilicity balance for a set of bRo5 compounds with respect to their absorption routes.[40] The position of ARV-110, ARV-471 and DDT2216 positions is highlighted. Other benchmarking compounds are presented in italics (macrocycles) and regular font (PROTACs). The x value of DT2216 is presented as relative since the Δ log *k*_w_^IAM^ descriptor is inaccurate for ampholytes.

As proven earlier [40], BRlog*D* and log *k*_w_^IAM^ are useful descriptors to simultaneously monitor the lipophilicity/polarity balance of bRo5 compounds. In particular, this balance was proven helpful in rationalizing the absorption route of several bRo5 examples. We incorporated the experimental data of the three investigated PROTACs into the originally published plot [40] and observed that the trend is maintained (Figure 9). ARV-110 and ARV-471 occupy an intermediate position in terms of lipophilicity and polarity, aligning with other oral compounds. Notably, both are less polar and more lipophilic than ARV-825, another oral CRBN-based degrader. Furthermore, the high lipophilicity and the presumed low polarity of DDT2216 places it outside the delimited oral zone. These results provide further support for the assertion that the experimental measurement of molecular properties is a mandatory part of any bRo5 drug discovery program.

As already mentioned, it is essential to have simple computational tools that can predict molecular chameleonicity in early drug design to prioritize the synthesis of derivatives with the desired physicochemical profile. Thus, conformational sampling simulations in water and chloroform were conducted only for ARV-110 and ARV-471, as the complex ionization profile of DT2216 precludes this type of analysis.

**Figure 10.**
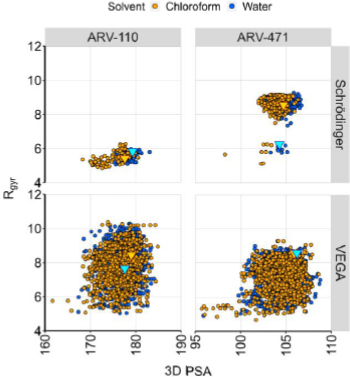
Chameleonicity prediction for the investigated compounds. The minimum energy conformers (MECs) are shown as triangles colored in gold (chloroform) and light blue (water). 3D strategies were not applied to DT2216 due to its complex ionization profile.

Two conformational sampling methodologies (MacroModel and VEGA ZZ) were evaluated. The former is a CS tool implementing the mixed model algorithm (MCMM/LMOD) already benchmarked for several PROTACs [48]. VEGA ZZ adopts an alternative conformational search method based on the “Boltzmann jump” method. Results are shown in Figure 10 (Figure 6 helps in the plot interpretation). ARV-110 and ARV-471 were analyzed and easily compared, given their shared E3 ligand-linker structure (Figure 7). The *R*_gyr_ *vs.* 3D PSA plots (Figure 10A) suggest that VEGA ZZ provides a generous number of conformers in aqueous and chloroform environments, but the environment-dependent differences are difficult to identify. MacroModel, on the other hand, provides solvent-dependent conformations with different polarity and shape characteristics for the two PROTACs. In fact, both compounds show less polar conformations in chloroform, which suggests a chameleonic nature. Moreover, ARV-471 is considerably more variable in terms of *R*_gyr_ than ARV-110, displaying a higher conformational variability, which could be related to the higher chameleonicity. The minimum energy conformers (MECs) do not provide additional information with either tool.

## Conclusions

New chemical modalities are gaining popularity in drug discovery due to their innovative mechanisms of action. However, their large and flexible bRo5 structures pose significant DMPK limitations. To address this challenge, recent literature emphasizes the need for efficient methods to quantify and optimize physicochemical properties when designing new oral bRo5 drugs. Achieving this goal requires working at multiple levels of complexity.

While 2D computed descriptors are easy to obtain, they fail to capture molecular properties influenced by conformation effects, such as IMHB formation. Therefore, their extensive application in advancing compounds through the bRo5 drug discovery pipeline should be approached with caution and critical analysis. A carefully chosen set of experimental physicochemical descriptors can be determined for bRo5 derivatives. These descriptors can effectively characterize the lipophilicity, polarity, and chameleonicity (the molecular property describing a compound's ability to undergo conformational changes to adapt to different environments) of bRo5 compounds. Chameleonicity can also be predicted using computational tools that generate conformers in diverse environments.

Combining these descriptors is anticipated to greatly enhance the efficiency of bRo5 drug discovery campaigns by offering robust filtering and prioritization strategies. However, some issues remain to be solved. This paper reports the full physicochemical characterization of three PROTACs in clinical trials. The data and their interpretation are robust for two of them, which can be considered predominantly neutral at pH 7. However, all the strategies encounter significant limitations with the third PROTAC due to its complex ionization profile. Research in this area is ongoing in our laboratory and, hopefully, in other labs as well.

## Methods

Experimental physicochemical data of the three PROTACs were obtained as reported in the original papers [23,40].

The computational study was performed using the following software. AlvaDesc (version 2.0.16, www.alvascience.com/alvadesc/), Vega ZZ (version 3.2.3, https://www.ddl.unimi.it/) and DataWarrior (version 06.01.00, https://openmolecules.org/datawarrior/) were used to calculate the 3D descriptors. The MacroModel plugin in the Maestro suite (Schrödinger, https://newsite.schrodinger.com/) and Vega ZZ were employed for the conformational analysis. The MacroModel methodology is reported elsewhere. [48] The Vega ZZ methodology is the following: first, the SMILES codes of the three PROTACs are converted into 3D structures with CORINA (https://demos.mn-am.com/corina_interactive.html) and then submitted to conformational sampling using the implemented AMMP program based on the SP4 force field and the Boltzman jump algorithm (temperature = 10000, number of minimized conformations =1000). Conformers are generated both in chloroform and in water by means of an implicit solvent model with a dielectric constant value of 4.18 and 80, respectively.
